# The effect of metal surface nanomorphology on the output performance of a TENG

**DOI:** 10.3762/bjnano.13.25

**Published:** 2022-03-15

**Authors:** Yiru Wang, Xin Zhao, Yang Liu, Wenjun Zhou

**Affiliations:** 1 School of Mechanical Engineering, Chengdu University, Chengdu 610100, China; 2 Institute for Advanced Study, Chengdu University, Chengdu, 610106, PR China; 3 Officers College of PAP, Chengdu, 610213, China; 4 School of Chemistry and Chemical Engineering, Neijiang Normal University, Neijiang 641100, China

**Keywords:** charge density, green energy, metal nanomorphology, triboelectric nanogenerator (TENG)

## Abstract

In this work, the effect of charge density and nanomorphology of a metal tip on the output performance of a triboelectric nanogenerator (TENG) is studied. The basic working principle of the TENG is charge transfer after separation of a metal and a polymer. There are different charge densities on different kinds of metal surface nanomorphology, which significantly influences the output performance of the TENG. Copper samples with different nanomorphology were obtained by controlling pH value, current density, electrolyte concentration, and temperature during the electrodeposition of copper. The samples were characterized using XRD and SEM. The output performance of the TENG is closely related to the size, charge density distribution, and shape of the metal nanoparticles.

## Introduction

Energy plays a vital role in human society. It is an important material basis for human activities and promotes scientific and technological development and economic growth. The current rapid economic development almost completely relies on non-renewable resources such as oil, coal, and natural gas. The consumption of fossil fuels, at the same time, causes many environmental problems. In this regard, looking for green renewable energies to replace fossil energies has become a future development trend. Especially the high degree of disorder that widely exists in nature and is not fully utilized has raised great interest of researchers [1–2].

**Figure 1:**
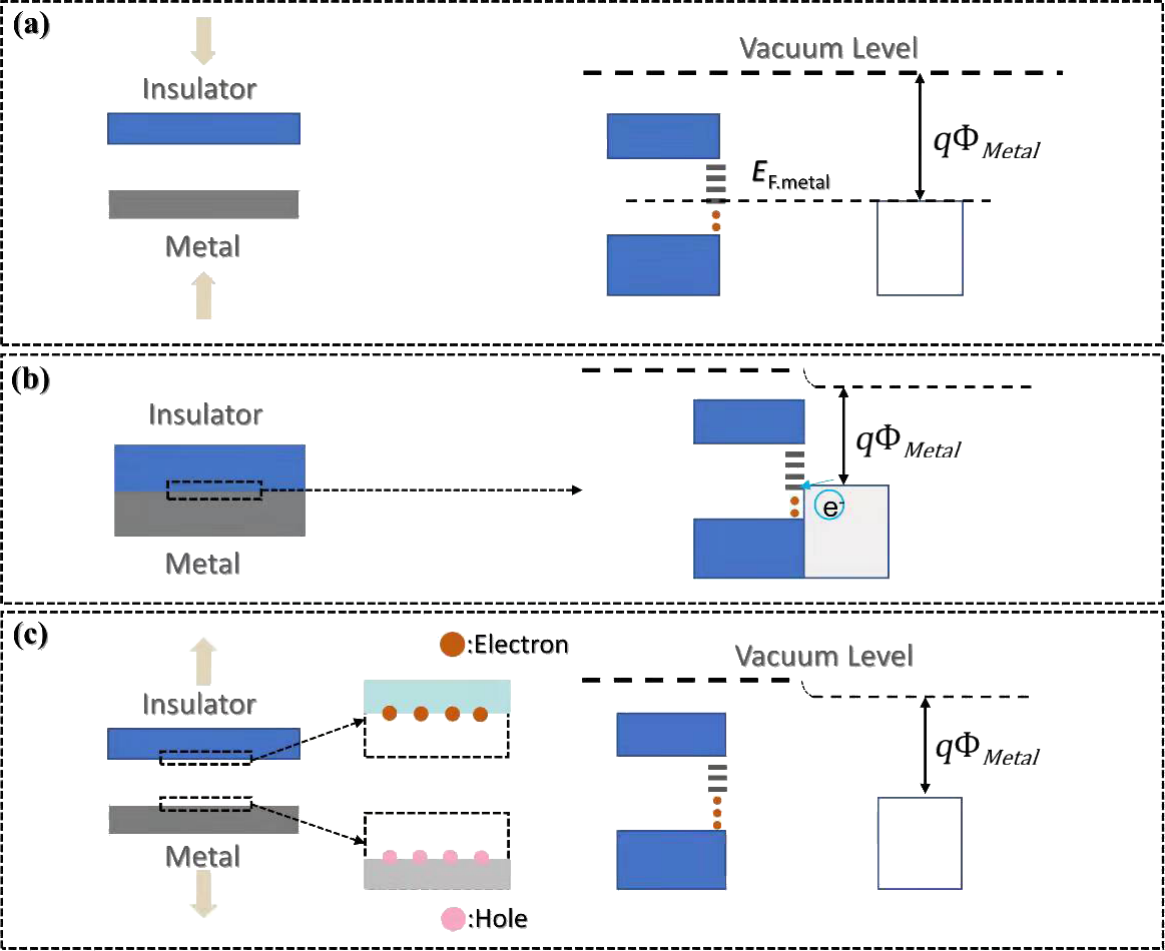
Energy band model of the metal–insulator contact electrification. The highest unoccupied intermediate trap state accepts electrons. Figure 1 was redrawn from [13] (ϕ_Metal_: metal work function).

Triboelectric nanogenerators (TENGs) are environmentally friendly energy collectors that improve energy utilization. They can use forms of renewable energy that are widely available in the environment and can replace non-renewable resources such as coal and oil [3]. In order to convert mechanical energy into electrical energy, various methods were developed, such as electromagnetic generators [4–6], piezoelectric materials [7–10], and pyroelectric materials [11–12]. The underlying principles of TENGs converting mechanical energy into electrical energy are the friction electrification effect and the electrostatic induction principle. When two materials with different electronegativity are physically contacted, positive and negative electrostatic charges are generated on each material surface. When the materials are separated, the positive and negative electrostatic charges on the materials will also be separated, resulting in a potential difference. The charge transfer strongly depends on the work functions of the two materials in contact, for example, metal–metal, semiconductor–semiconductor and semiconductor–metal contact pairs [14–15]. A semiconductor–metal contact can be described by the band diagram shown in Figure 1. The frictional electrical properties of materials depend on their work functions and Fermi levels [16–17]. The intermediate state in the bandgap can reduce the barrier of electron transfer, thus, enabling electron flow from insulator to metal (vice versa) or from an insulator to another insulator.

TENGs are miniaturized and portable. They generate current by collecting tiny amounts of energy and supply power for microelectronic devices and sensors. Wind energy [18–21], water wave energy [22–23], acoustic energy [24–25], and energy generated by human activities [26–27] can be collected and effectively converted into electrical energy for medical, meteorological, and other applications. A new type of TENG with environmental protection, high reliability and low cost is being actively developed [28–32]. However, because of the small size of TENGs, the output performance is greatly limited. Improving the output performance is a factor that must be considered when designing and manufacturing TENGs.

The selection of materials plays an important role regarding the output power of TENGs. The selection of appropriate materials can significantly improve the amount of transferred charge. Basic physics explains the difference in the amount of transferred charge between different materials. In addition, after TENGs were invented in 2012 [33–34], many attempts have been made to enhance the energy harvesting efficiency. There are four ways to enhance the efficiency of energy collection. These are (1) surface treatment of the contact materials, including increasing the surface roughness and physical surface modification to enhance the surface charge density [35–37], (2) reduction of the impact of the external environment on TENGs [38–39], (3) enhancement of the surface charge density, including active charge pumping and intercalation of a charge trap layer [40–41], and (4) increase of the number of TENG units [42–43].

In order to improve the surface charge density in the contact electrification process, it is necessary to expand the effective contact surface area by surface engineering of micro-/nanoscale structures [44–46]. There are various processes for the surface engineering of polymers with micro-/nanoscale structures, such as template forming, plasma treatment, and chemical approaches. Previous studies mostly focused on the preparation process of the nanoscale morphology on the polymer surface [47–49]. The electrons on the polymer surface cannot be transferred to the conduction band and the charge cannot flow freely. Therefore, there is no high charge density on the tip surface of the polymer surface.

Here, size and morphology of nanoscale copper were controlled by adjusting current density, temperature, pH value, and solution concentration during electrodeposition. The effects of different morphologies and sizes on the energy harvesting efficiency of the TENG were studied. The enhancement effect of different surface charge density distributions on the output performance of the nanostructured metal TENGs is explained.

## Experimental

### Materials and characterization

In this study, 1 M sulfuric acid, copper sulfate pentahydrate (purchased from Yongrong) with purity greater than 99.5%, and deionized water were used to prepare the electrolyte solution. The experimental temperature was controlled using a water bath (Olabo, model HH-S6). A Kelong KLX305 DC power source was used to control the current density during electrodeposition. The sample after electrodeposition was dried in a Geruida GRD220H oven.

After obtaining 16 groups of experimental samples, PTFE (purchased from Bukraun) was used as the anode, and the experimental sample was used as the cathode to fabricate the TENGs. The open-circuit voltage was measured using an oscilloscope DS1102E (produced by Rigol). The short-circuit current was tested using an electrochemical workstation (CH, model CHI660E). The crystal structure of the samples was analyzed using a Bruker D8 Advance X-ray diffractometer. A scanning electron microscope (Coxem, model EM-30) was used to observe the nanocrystalline structure of the samples in detail.

### Fabrication

#### Fabrication

The copper surface was pretreated by polishing the copper sheets with sandpaper of 1000, 3000, and 5000 mesh. Subsequently, the copper was acid deoiled to remove organic stains on the surface and copper oxide was activated at the same time. After acid oil removal, the copper sheets were washed with deionized water at 25 and 50 °C, and the residuals from the previous process were cleaned to avoid polluting the electroplating solution. Different pH values were set using 1 M sulfuric acid. Copper sulfate pentahydrate of different concentrations (50 mL) was added to the electroplating solution.

The electroplating process was based on an orthogonal test design for 16 sets of experimental conditions. First, the electrolytic cell was filled with electrolyte and the pole plate was immersed into the electrolyte. The deposition area was set to 9 cm^2^ by adjusting the position of the anode copper sheet and the cathode copper sheet immersed in the electrolyte. The distance between the anode and cathode plates was fixed at 5 cm to avoid the impact of plate spacing on the nanomorphology of the electrodeposited copper. The current was set according to the required current density. The plating solution was heated to a fixed temperature using a water bath, and the electrodeposition was carried out for a period of 30 s.

The influence of the nanomorphology on the output performance of the TENG was assessed through the “contact and separation” method. One cyclic period of contact and separation was set to 1.5 s (contact of 0.75 s and separation of 0.75 s). The applied force from the mechanical shaker was fixed at 100 N. Output voltage and current were measured using a digital oscilloscope and an electrochemistry workstation.

#### Orthogonal experimental design

The copper nanomorphology was controlled by variation of solution concentration, pH value, current density, and temperature. The current density affects the speed of electromigration. The orthogonal test (Table 1) was designed regarding four parameters, that are, electrolyte concentration, current density, pH value, and temperature. The experimental parameters for all 16 samples are shown in Table 1. The concentration of CuSO_4_·5H_2_O was set between 0.25 and 1 M, the temperature was set between 25 and 85 °C, the current density was set between 50 and 500 A/m^2^, and the pH value was set between 1 and 4.

**Table 1:** Experimental parameters for the copper deposition.

Sample	CuSO_4_·5H_2_O concentration (mol/L)	Current density (A/m^2^)	pH value	Temperature (°C)

1	0.25	50	1	25
2	0.25	100	2	45
3	0.25	300	3	65
4	0.25	500	4	85
5	0.5	50	2	65
6	0.5	100	1	85
7	0.5	300	4	25
8	0.5	500	3	45
9	0.75	50	3	85
10	0.75	100	4	65
11	0.75	300	1	45
12	0.75	500	2	25
13	1	50	4	45
14	1	100	3	25
15	1	300	2	85
16	1	500	1	65

**Figure 2:**
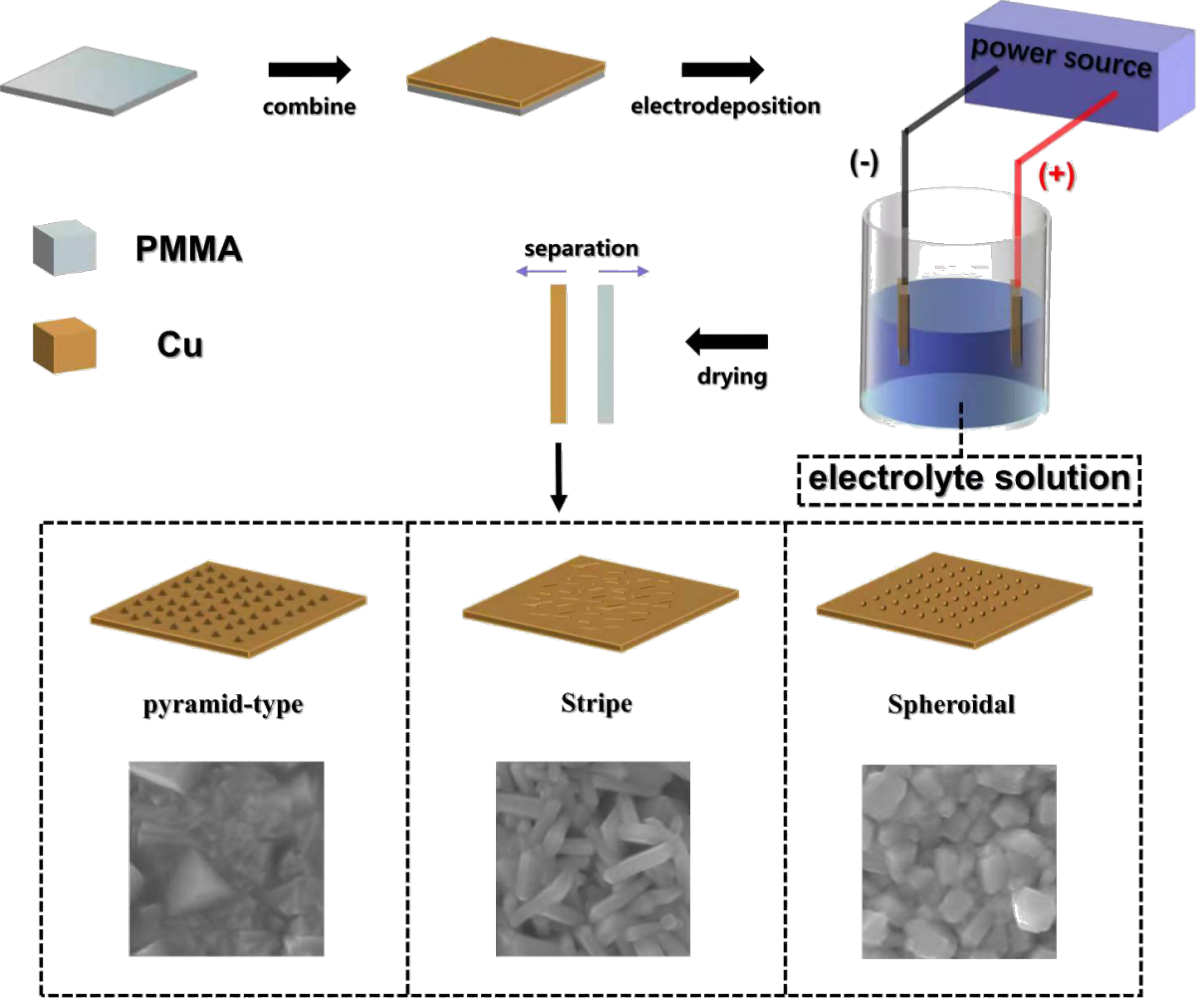
Electrodeposition process. A PMMA plate was used as the electrodeposition liner.

When copper and PTFE film are in contact, the surfaces of the two materials are charged. When the two materials are separated, the surfaces of the two materials will maintain the charge, and a potential difference will occur between the two materials. Hence, electric charges can move from one electrode to the other through an electrostatic field. When the materials are brought in contact again, the electrostatic field will disappear. Finally, the electrons flow in the opposite direction. An alternating current will be generated through the repetition of this process. After 30 s of deposition under different conditions, different morphologies of nanocrystals, including pyramids, strips, and spheroids, were obtained (Figure 2).

**Figure 3:**
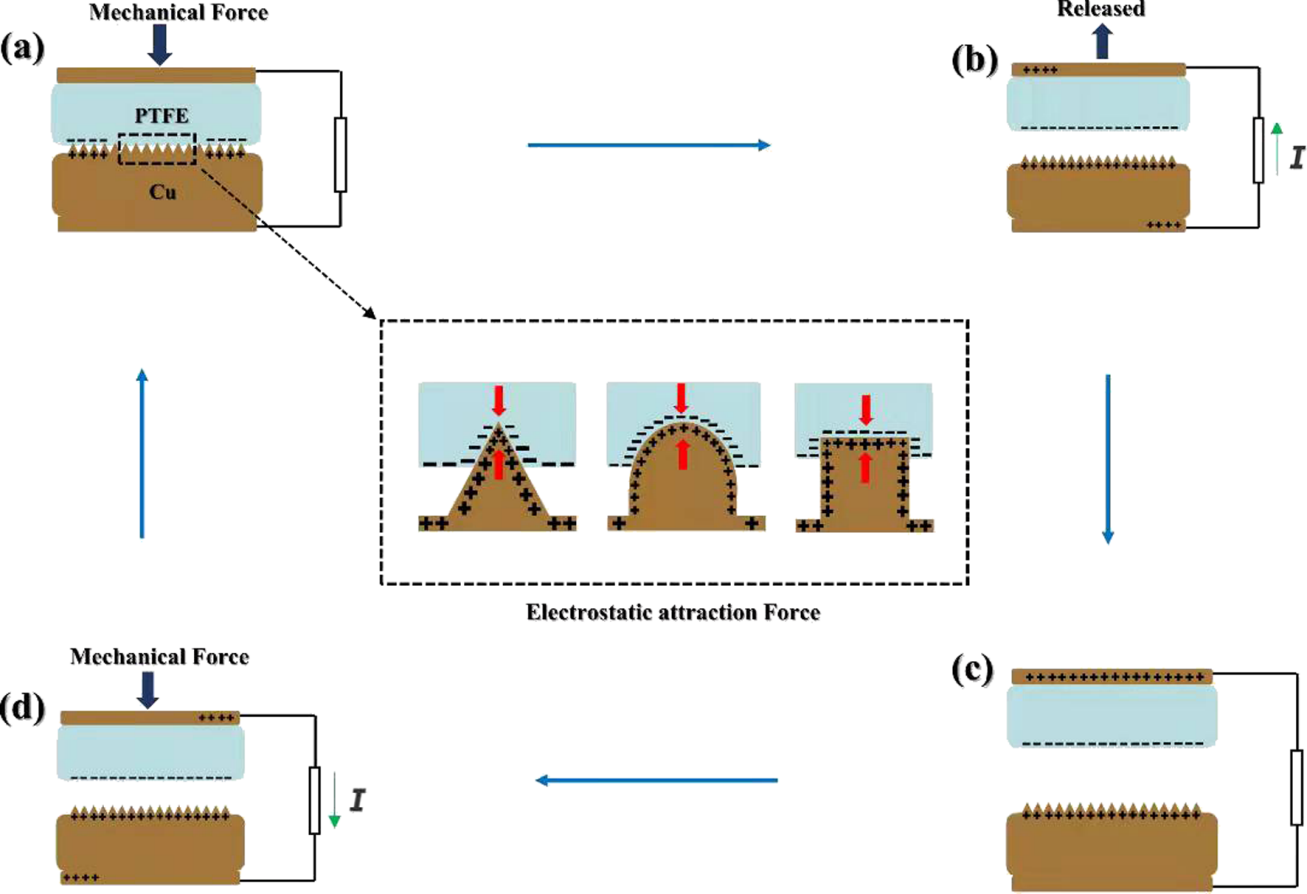
Contact and separation of Cu and PTFE.

At the beginning of the experiment, Cu and PTFE are in contact through an external force. The surface charge on Cu is positive, and that on PTFE is negative (Figure 3a). A separation is caused by the removal of the external force, and electrons flow from the PTFE electrode to the Cu electrode (Figure 3b). Then, charge exchange is carried out at the contacts. Electrical equilibrium is formed when the Cu and the PTFE are separated by a greater distance (Figure 3c). When the external force is applied again to bring Cu and PTFE into contact, electrons will flow from the Cu electrode through an external load to the PTFE electrode (Figure 3d).

According to the observation of the diameter of the nanoscale blocks grown in the above experiments, it can be concluded that at high temperatures and small pH values, the copper nanoscale topography grows more finely. When solution concentration and current density are high, and when the flow rate of the plating solution is low, the electrodeposited copper nanoscale structures will agglomerate.

## Results and Discussion

**Figure 4:**
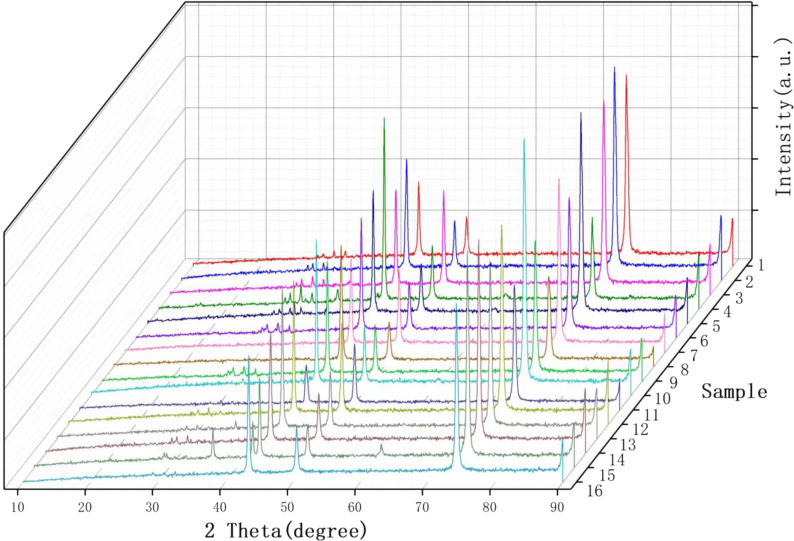
XRD diffraction patterns of the 16 samples after electrodeposition.

**Figure 5:**
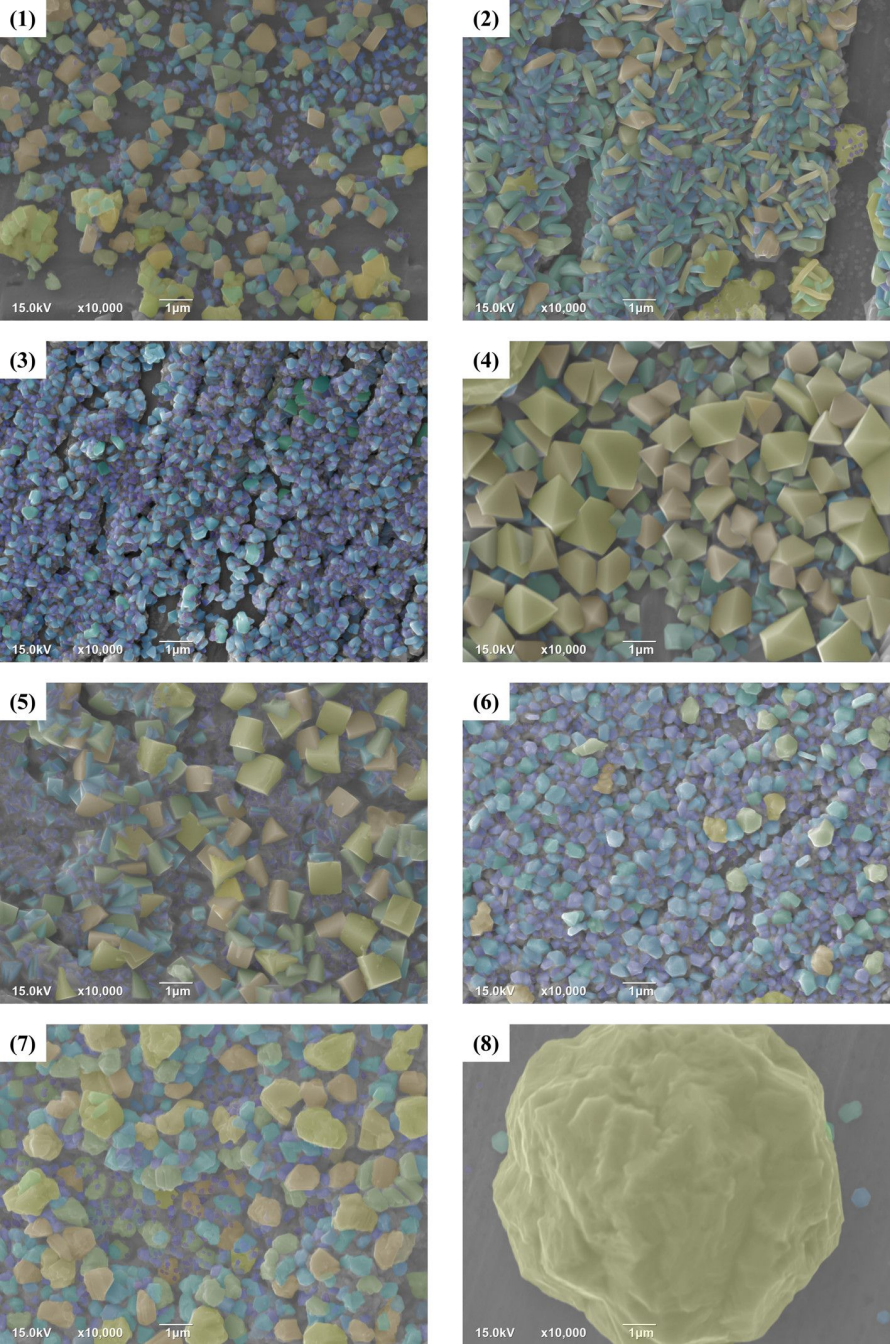
Samples 1–8 of the 16 samples were screened and classified according to the particle size. Different colors were selected for different particle sizes to visually display the particle size distribution. Samples 4 and 5 show pyramidal nanostructures, samples 3, 6, 7, and 8 show spheroidal nanostructures, and samples 1 and 2 show nanoscale strips.

**Figure 6:**
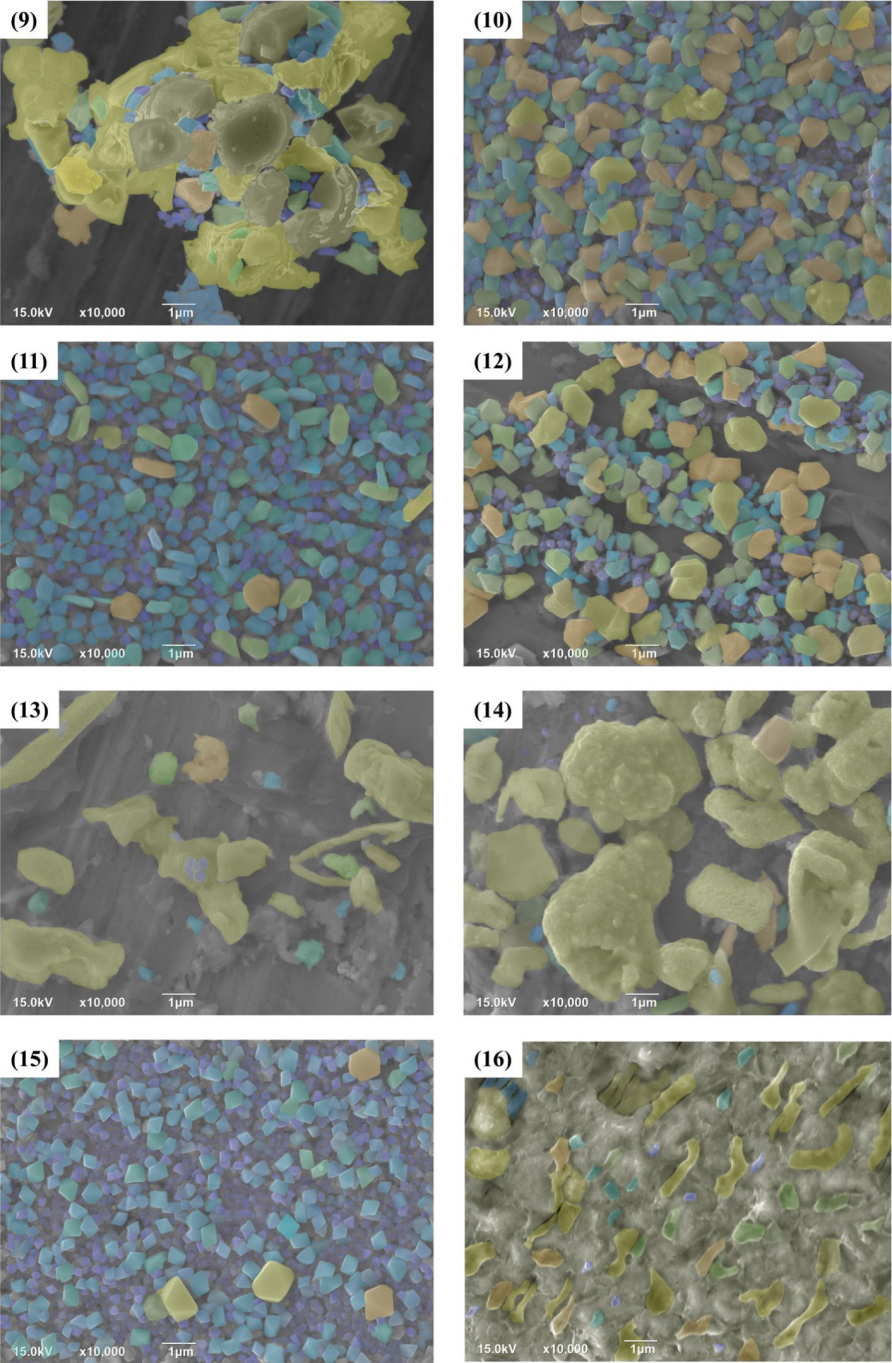
Samples 9–16 of the samples were screened and classified according to the particle size. Different colors were selected for different particle sizes to visually display the particle size distribution. Samples 15 shows pyramidal nanostructures, experiments 9, 11, and 12 show spheroidal nanostructures, and samples 10, 13, 14, and 16 show nanoscale strips.

The XRD data (Figure 4) were processed using the JADE software to calculate the average particle sizes. SEM micrographs were screened according to the surface morphology size of the nanoparticles and colored according to the nanoscale topography size (Figure 5 and Figure 6). After the size screening, the proportion of each particle size was calculated. The particle size distribution plays a vital role in improving the output performance. The nanocrystals can be divided into pyramids, spheroids and strips. The shape of the metal nanocrystals determines the distribution of surface charge density, which, in turn, influences output current and voltage.

**Figure 7:**
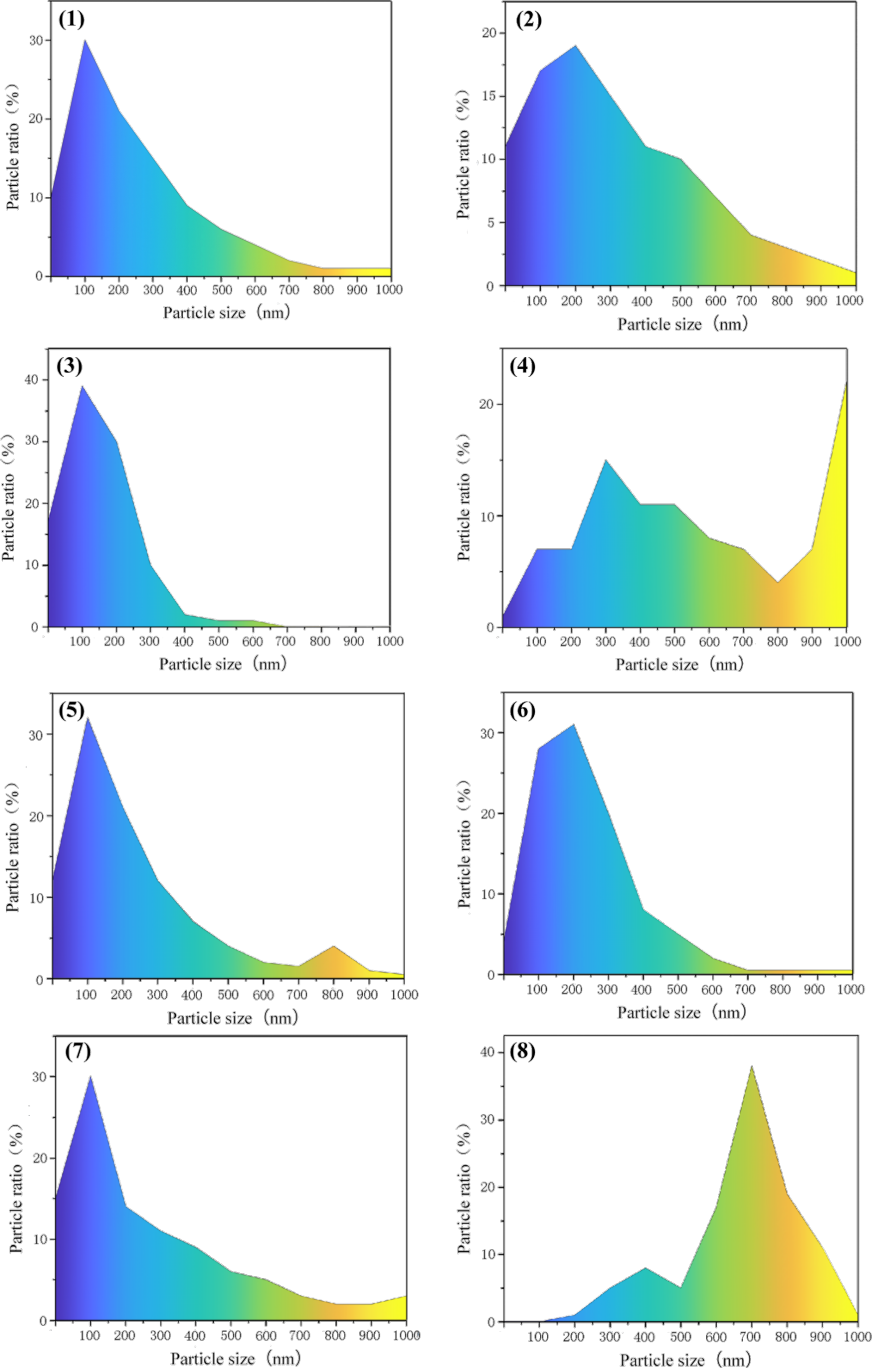
The particle size distribution of samples 1–8 of the 16 copper nanostructures.

**Figure 8:**
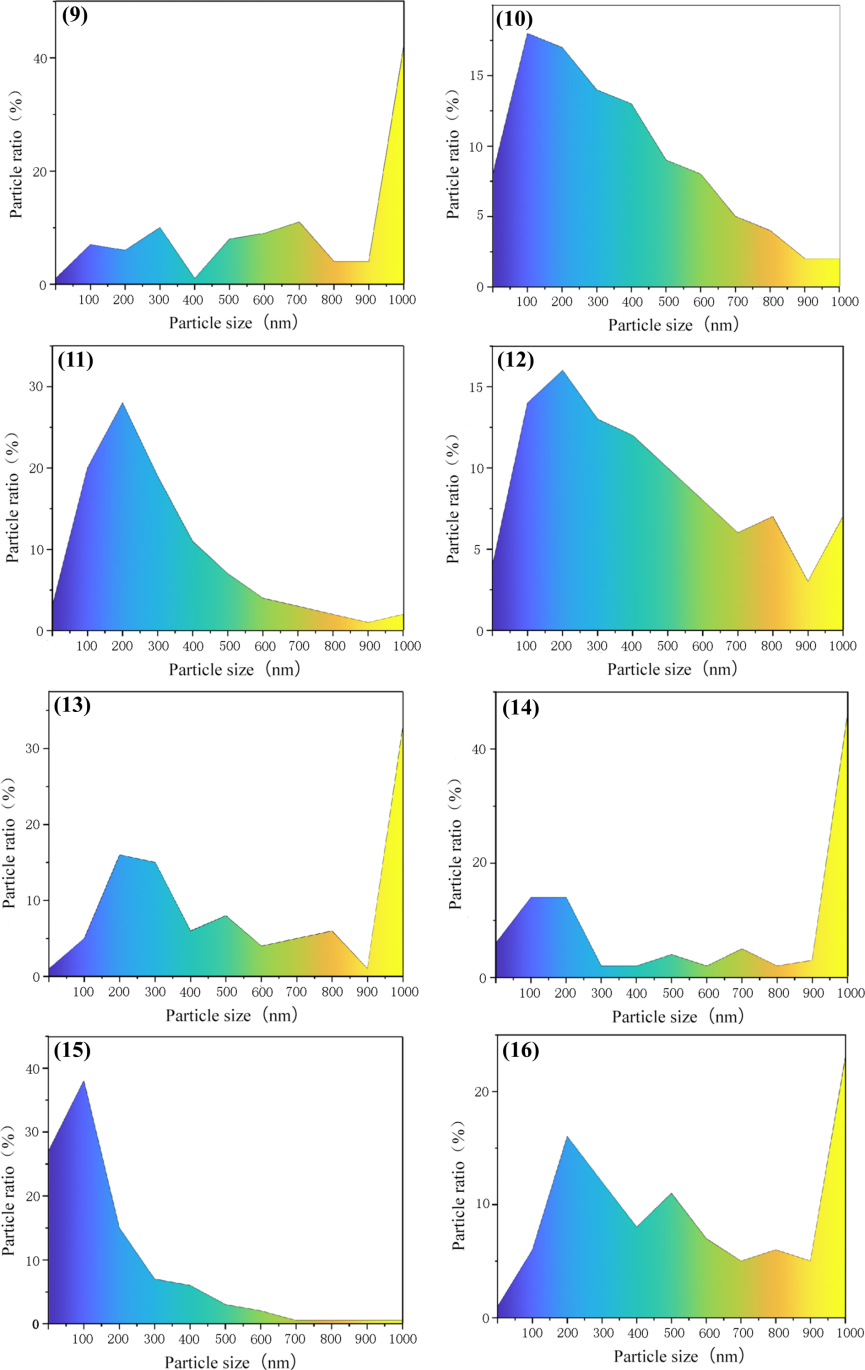
The particle size distribution of samples 9–16 of the 16 copper nanostructures.

Size and shape of the particles are related to the performance of TENG. The particle size distributions are given in Figure 7 and Figure 8. Narrower size distributions of copper nanoparticles lead to a higher output performance. Larger size distribution gaps lead to weaker output performance. The charge density of the metal surface is inversely proportional to the curvature radius of the metal surface, that is, the sharper the metal surface, the greater the surface charge density in the sharp part.

**Figure 9:**
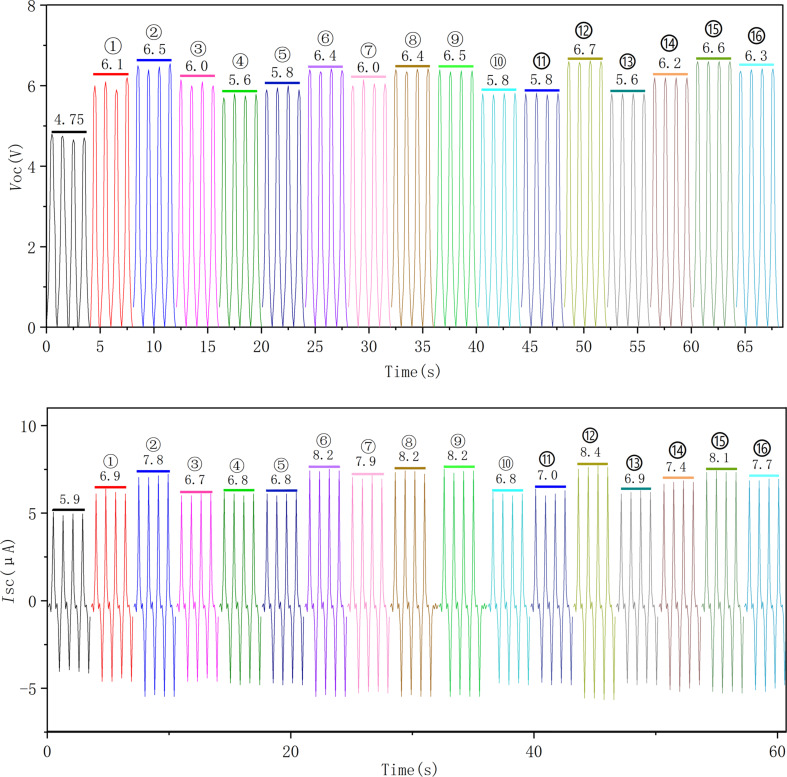
The output performance of the 16 samples. (a) Open-circuit voltage and (b) short-circuit current.

In sample 4 (0.25 mol/L, 500 A/m^2^, pH 4, 85 °C), the proportion of large particles (≥1000 nm) is 22%, and the proportion of small particles (100 to 400 nm) is 30%. When large and small particles coexist, the improvement of the output performance is only 18% (Figure 9). In contrast, small particles accounted for 62% and large particles accounted for 1% of the particles in experiment 2 (0.25 mol/L, 100 A/m^2^, pH 2, 45 °C). The output performance was 6.5 V, which was 37% higher than that of a copper sheet without nanoscale topography (Figure 9). The output performance of experiments 6, 12, and 15, was improved by 35% to 40% (Figure 9). Generally, due to the increase of contact area and surface charge density a higher friction electrical output is produced. Large copper nanoparticles have full contact with the polymer, but small copper nanoparticles have insufficient contact with the polymer or even no contact at all. Therefore, the improvement of friction electrical output performance is not obvious.

Experiments 4, 5, and 15, which are shown in Figure 5 and Figure 6, respectively, yielded pyramidal copper nanoparticles with sharp surfaces. Among them, experiments 4 and 5 improved the output performance only by 18% to 19%, and the improvement is not obvious (Figure 9). This is because the particle size distributions are too large. This can be seen intuitively in the COMSOL displacement simulation below in Figure 11. However, the charge density at the sharps tips is higher than that at the spherical strips, which is very obvious in experiment 15. The particle size distribution of experiment 15 is relatively concentrated, and its improvement on the output performance reaches 34%, almost twice that of experiments 4 and 5 (Figure 9).

We measured the nanoparticle size and its variance and studied the relationship between the variance and the voltage; the more dispersed the particle size, the smaller the improvement of the output efficiency, the less dispersed the particle size, the greater the improvement of the output efficiency. The reason for this phenomenon is significant size gaps, that is, the large nanoparticles are in full contact with PTFE, but the small nanoparticles have no or not full contact with PTFE, which leads to the difference in output efficiency.

**Figure 10:**
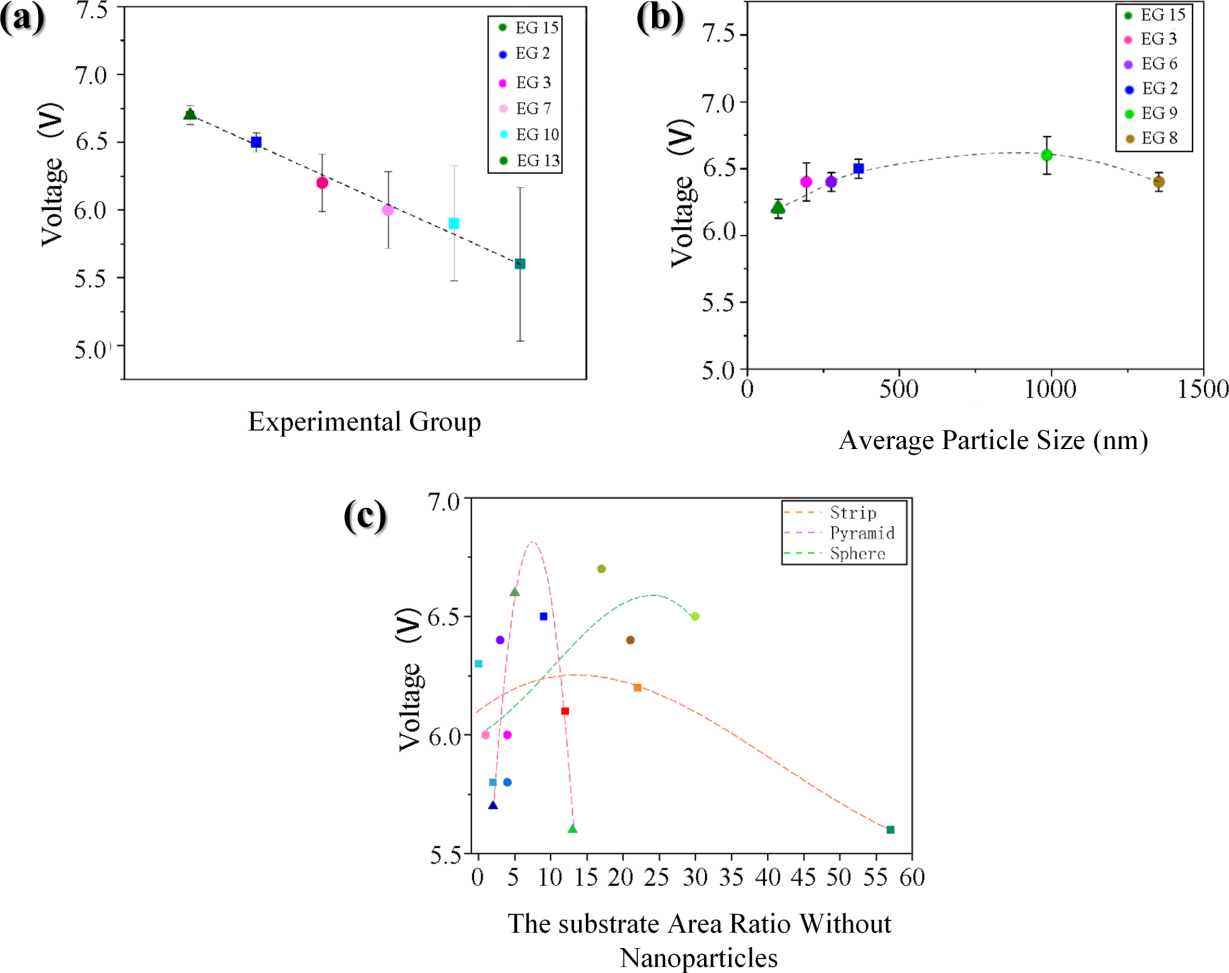
(a) Effect of particle size variance on the open-circuit voltage (EG = experimental group = sample, as listed in Table 1). (b) Influence of average particle size on the open-circuit voltage in a narrow size distribution. (c) Effect of grain distribution density on the output performance according with different shapes of nanoscale crystallites.

The effect of surface topography on the output performance was studied by classifying the nanocrystals according to different shapes. The reason for the influence of the abovementioned variance on the output performance and the influence of shape on the output performance is that the surface charge densities of the different nanoscale copper surface topographies are different. The sharper the surface is, the higher the charge density is. Combined with the above process of electron exchange after the contact between metal and insulator, the output performance can be effectively improved. Figure 10 shows the effect of the grain density distribution (based on the grain shape) on the output performance of the TENG. Because the difference of granularity variance will also greatly affect the output performance, especially in 15 out the 16 samples, the output performance significant improved due to narrow particle size distribution.

We also studied the effect of the average particle size on the output efficiency. The average particle size data were obtained from the JADE software as mentioned above. It can be seen from the colored SEM images that when the particle size distribution is narrow, the average particle size has no obvious effect on the output efficiency, and the contact area between large particles and small particles and the PTFE polymer is not reduced.

Subsequently, the SEM images were analyzed and processed by using the Matlab edge detection algorithm, and the IMOVERLAY script in Matlab File Exchange was used for pixel analysis. Thus, the percentage of the substrate area without metal nanoparticles was calculated by the percentage of pixel filling. The effect of particle density on the output performance was studied (Figure 10c). It can be seen from the fitting curve that the output performance increases with the increase of the substrate area fraction without nanoparticles, but when the substrate area fraction without nanoparticles is greater than 30%, the output performance will be significantly reduced. The reason for this phenomenon is that the output power of a TENG is related to the charge retention between the gaps [50]. The charge is transferred when the polymer is completely in contact with the copper nanoparticles. There is no potential at this stage, so the transferred charge will accumulate in the gap of metal nanoparticles, and the charge accumulation will have a positive impact on the output performance. However, when the substrate area without nanoparticles is too large, the decrease in the number of metal nanocrystals will directly affect the contact area and the output performance will be greatly reduced.

**Figure 11:**
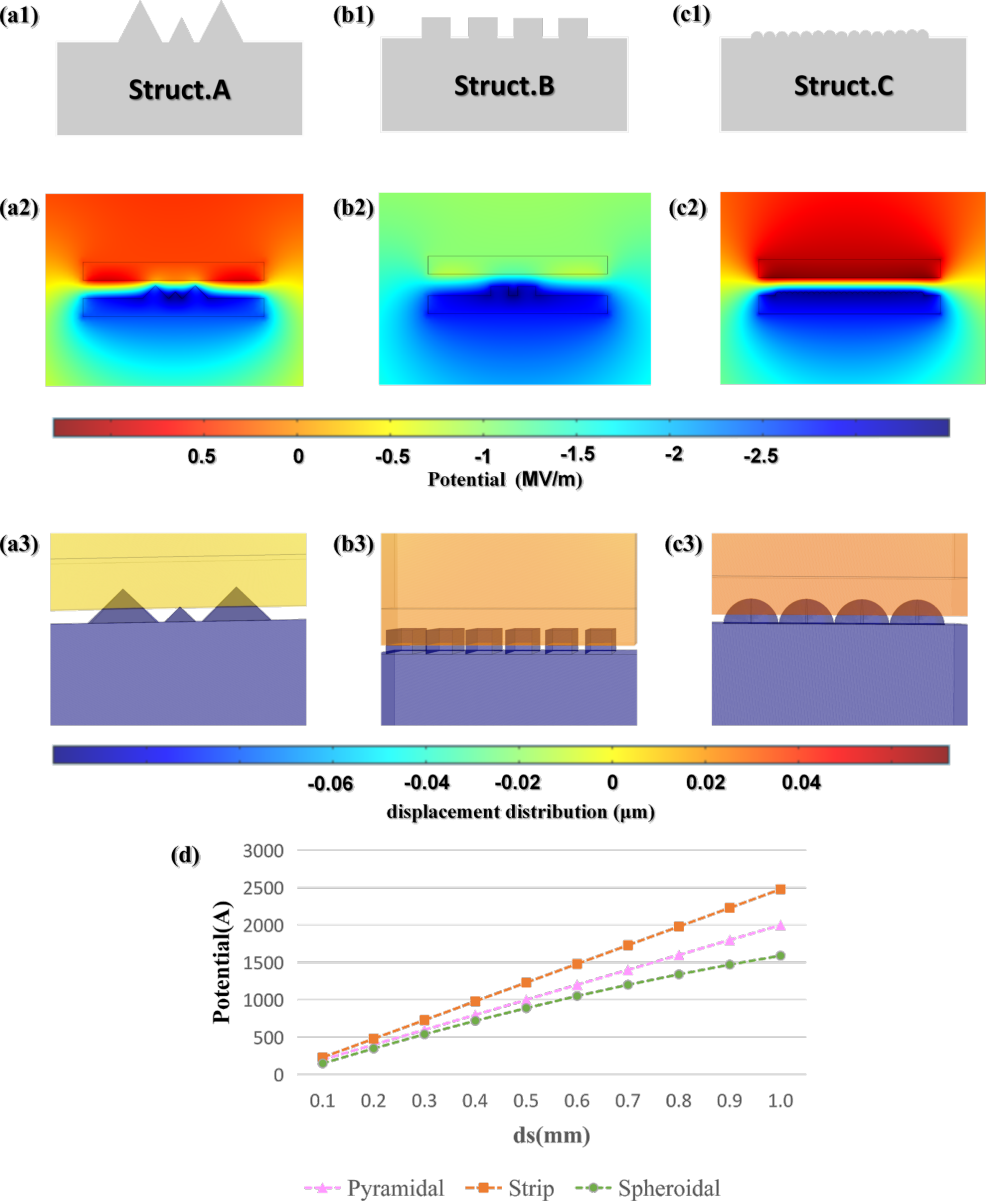
(a1–c1) Models of pyramids, strips, and spheroids. (a2–c2) COMSOL simulation of the electric field distribution with a surface charge density of 12.5 μC·m^−2^ at the contact surface of PTFE. (a3–c3) COMSOL simulation of the displacement distribution under a pressure of 3 MPa. (d) Potential of the three TENG shapes considered here.

Nanostructured surfaces with different particle size distributions were prepared under different experimental conditions. According to the particle shapes, the nanostructures have been divided into three types labeled “Struct. A” (pyramidal), “Struct. B” (strips), and “Struct. C” (spheroidal) (Figure 11). A COMSOL simulation can explain the differences in output performance caused by the different particle shapes. Taking pyramidal structures as an example, it can be seen in the simulated displacement image that when a pressure of 3 MPa is applied to the PTFE surface at the top of the Cu nanoparticles, the surface structure of the PTFE polymer is bent (Figure 11). The large nanostructures on both edges are in contact with the polymer surface, but the small nanostructure in the middle is not in contact with the polymer. A two-dimensional model (modeled according to the size distribution) was created to represent different surface morphologies. In addition, the potential distribution of different shapes was simulated, and different surface charge densities of the different shapes are given. It can be concluded that nanocrystal strips can yield a good electron output. Due to the different surface contact charge densities of the copper nanoscale morphology, under pressure, the curved PTFE structure transfers more friction charges, which contributes greatly to improving the output performance.

## Conclusion

In this paper, distribution density, average particle size, and size variance distribution of nanoparticles have been studied. It can be concluded that the distribution density of nanoparticles has a negative correlation with the improvement of TENG output, but when the distribution density becomes very sparse, the output performance decreased rapidly. The average particle size hardly influences TENG output performance. In contrast, the grain size variation has a significant impact on the improvement of the output performance. When the grain size variance is very large, the increasing rate of the output performance sinks. A simulation shows that nanoscale strips yield an optimal output performance, followed by pyramidal shapes.

In addition, the electrodeposition rate of copper and the size of electrodeposited copper nanocrystals can be adjusted via the current density. The temperature has an appreciable influence on the mass transfer of Cu^2+^ ions in the plating solution, which affects the growth of electrodeposited copper nanoparticles. Temperatures that are too high will increase the total resistance of the system. The concentration of copper ions has a decisive effect on the growth density of the copper nanocrystals. These conclusions may help in improving the output performance of TENGs through the control of the metal surface morphology.
